# Cervical Oculopathy: The Cervical Spine Etiology of Visual Symptoms and Eye Diseases—A Hypothesis Exploring Mechanisms Linking the Neck and the Eye

**DOI:** 10.3390/diagnostics15202650

**Published:** 2025-10-21

**Authors:** Ross A. Hauser, Morgan Griffiths, Danielle Matias, Benjamin R. Rawlings

**Affiliations:** 1 Caring Medical Florida, 9738 Commerce Center Court, Fort Myers, FL 33908, USA; rhprolobook@caringmedical.com (R.A.H.); steilend@caringmedical.com (D.M.); 2 Independent Researcher, Fort Myers, FL 33908, USA; morgriffiths@gmail.com

**Keywords:** cervical vertebrae, joint instability, diagnostic imaging, ultrasonography, optic nerve, vision disorders, cerebrospinal fluid, vagus nerve injuries, jugular veins, intraocular pressure

## Abstract

**Background:** Eye and visual symptoms are becoming increasingly common in young people, along with the emerging conditions text neck and computer vision syndrome, though underlying mechanisms are not fully elucidated. The link between cervical spine structure and the eye remains relatively unexplored. **Methods:** This paper employs a hypothesis-driven, literature-based evidence approach, aiming to explore the hypothesis that cervical spine structural issues may be an underlying mechanism for visual symptoms and eye diseases. The purpose of exploring this hypothesis is to lay the groundwork for future research, and advance diagnostics and treatment options. No new analysis was performed. **Results:** This article lays the groundwork for the hypothesis that cervical spine structural dysfunctions, including a forward-displaced atlas (C1), can cause dynamic carotid sheath compression, contributing to neurological and neurovascular mechanisms that affect the eye, primarily by (1) impaired venolymphatic drainage of the eye and brain due to compression of the internal jugular veins, and (2) ocular dysautonomia from a disruption of the parasympathetic/sympathetic system balance, partly due to vagus nerve degeneration. **Conclusions:** Potential mechanisms, diagnostics, and treatment options for visual disorders initiated by cervical structural dysfunction are discussed, providing a foundation for future research aimed at improving clinical outcomes for some eye conditions which have an otherwise unknown etiology.

## 1. Introduction

Lifestyle is a recognized, modifiable risk factor for the more than 2.2 billion people who live with visual impairments worldwide, and our modern lifestyle heavily depends on computers and cell phones [1,2]. Globally, macular degeneration and glaucoma are leading eye conditions which, according to the WHO report, are expected to continue rapidly increasing from 195.6 million to 243.4 million, and 76.0 million to 95.4 million, respectively, from 2020 to 2030 [3]. There is a high prevalence of visual disorders in young people, with children and adolescents approximated to make up 1/3 of the global prevalence of myopia, and 80% of the global prevalence of computer vision syndrome in the age range of 9.7 to 54.7 years [4,5]. A consistent finding for this young age group is the link between excessive screen time and visual disorders, but without clear mechanisms [6,7]. Eye and visual symptoms and diseases greatly affect the quality of life and overall health of a person, highlighting the importance of advancing diagnostics and treatment options [8].

This paper explores the hypothesis that an overlooked cause of eye diseases and visual symptoms may be structural neck issues. As blood, lymph, cerebrospinal fluid, and numerous nerves pass through the neck, undiagnosed and progressive degenerative neck conditions could be a missing link to improving patient outcomes, especially in patients who are otherwise unresponsive to traditional treatments [9].

Some eye and visual symptoms and conditions including—but not limited to—blurry vision, visual distortions, eye pain, oscillopsia, gaze instability, eye tearing, dry eyes, optic neuropathy, macular degeneration, retinopathy, cataracts, and glaucoma could originate from structural neck issues [10]. While the term “cervicogenic visual dysfunction” denotes cervical spine issues that can cause blurred vision or distorted vision, cervical oculopathy is a more encompassing term [9]. “Cervical” means pertaining to the neck, and “oculopathy” is a general term for eye diseases; thus, cervical oculopathy (COP) describes eye diseases and pathology coming from disorders in the neck.

The framework discussed here posits that the main etiology of COP, especially in apparently healthy young people, is ligamentous cervical instability (LCI), a detrimental effect on the cervical spine often “simply” due to the modern lifestyle that involves prolonged poor head and neck postures while using electronic devices. *Ligamentous cervical instability is the inability of the cervical ligaments to maintain proper alignment of individual, adjacent, or all cervical spine vertebrae when subjected to increased forces from various postures, positions, and movements, affecting the bones, soft tissues, and neurovascular structures, leading to symptoms* [11].

LCI and cervical dysstructure (CD) can be documented by digital motion (fluoroscopic) X-ray and upright cone beam CT scan [12]. Upper cervical facet joint (C1–C2) ligamentous instability is easily seen on digital motion X-ray as excessive movement of the atlas (C1) in relation to the axis (C2) in the lateral plane [12]. The pathophysiology it causes, including dynamic carotid sheath compression, can be evaluated by an in-office noninvasive testing, termed “neck vitals analysis.” [13]. The diagnostic testing process described can also be repeated to show improvements in cervical neck structure and the pathophysiology it causes. If it is found that some eye and visual symptoms and diseases have a cervical structural dysfunction as their root cause, then improvements in cervical curve and stability should be considered as part of a comprehensive approach to treatment of the disorders. Future prospective studies are warranted to assess the long-term outcomes of such an approach. If improvement in cervical structure correlates with symptomatic improvements in eye and visual symptoms in larger prospective studies, then the hypothesis of this paper will be validated.

**Figure 1:**
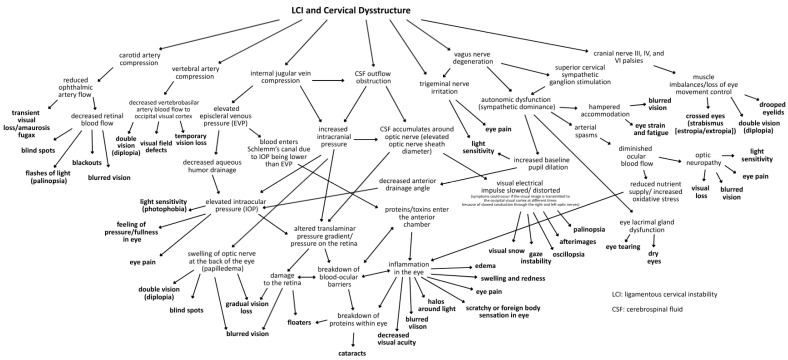
Conceptual framework mapping the many hypothesized mechanisms linking visual symptoms and eye diseases to LCI and cervical dysstructure [14,15,16,17,18,19,20,21,22,23,24,25,26,27,28,29,30,31,32,33,34,35,36,37,38].

The purpose of this paper is to introduce the novel hypothesis that cervical structural issues such as ligamentous cervical instability can be an overlooked cause of ocular conditions primarily due to carotid sheath compression, which impairs venous drainage and disrupts autonomic regulation of the eye, to lay the groundwork for advancing diagnostics and treatment options. This paper outlines the many ways the breakdown of the cervical structural support, especially LCI, could affect the visual system and how this condition and the pathophysiology it causes can be objectively measured and potentially treated. While this hypothesis paper mainly focuses on discussing carotid sheath compression (IJV and vagus nerves), additional mechanisms may also play a role (Figure 1).

## 2. Methods

This paper employs a hypothesis-driven, literature-based evidence approach aiming to explore the hypothesis that cervical spine structural issues may be an underlying mechanism for eye and visual symptoms and diseases. The methods consisted of identifying, reviewing, and synthesizing relevant published evidence to support the development of the proposed hypothesis, with the goal of generating a framework for future studies. No new statistical analysis was performed. This approach is intended for hypothesis generation and the mechanisms discussed are based on the theoretical integration of existing evidence. Studies that provided insights from various disciplines, including ophthalmology, neurology, anatomy, physiology, clinical sciences, and more, were integrated based on their relevance to the mechanisms under consideration.

## 3. The Modern Lifestyle’s Effects on the Cervical Spine

### 3.1. Facedown/Forward Head Lifestyle and Global Increase in Visual Impairment

It is estimated that by 2050, more than 20 million individuals in the U.S. will be affected by visual impairment—double the amount of people affected in 2015 [39]. There has been a reported 91.46% global increase in visual impairment from 1990 to 2019, with about 2.2 billion people suffering from visual impairments worldwide in 2020 [1]. Lifestyle changes, including more time spent indoors and on electronic devices, are suspected to be major contributors to the drastically rising number of visual impairments, but without a clear consensus on the reasons why, lending support to this hypothesis that eye and visual symptoms can have an underlying neurologic or neurovascular pathology due to changes in the cervical spine [40].

The increased reliance on digital devices is accompanied by an excessive amount of time spent in unnatural postures. Currently, one of the most prevalent yet often underappreciated postural abnormalities of the cervical spine is forward head posture (FHP) [41,42]. FHP is an emerging term used to describe the forward displacement of the head in comparison to the trunk of the body, putting strain on the neck and stretching the vital neurovascular structures and tissues [43].

In 2021, Americans averaged over 8 h a day using digital media [44]. Several studies have linked prolonged screen time to poor posture and resulting symptoms, but there is a missing explanation for the resultant symptoms such as eye pain/fatigue, headache, dizziness, and dry eyes [45,46,47]. Some researchers are already questioning whether smartphone overuse leads to ocular symptoms like myopia, blurred vision, and poor vision, and are recommending limited usage, but typically only consider the effects of blue light and eye strain [48].

**Figure 2:**
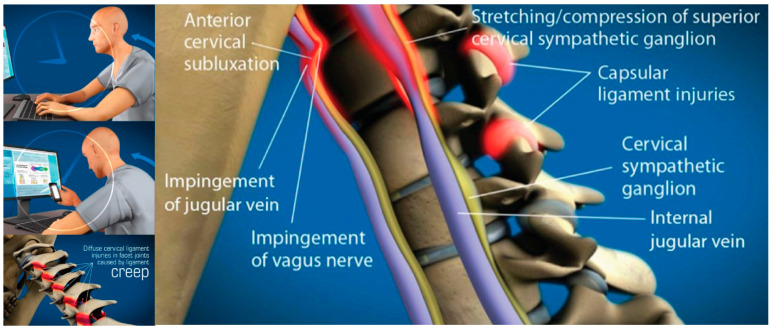
Forward head posture resulting in cervical ligament laxity, a progressive condition ultimately compressing the internal jugular vein and vagus nerves. “Creep,” which is a term signifying the slow stretching of ligaments, can occur in the cervical spine by a forward head posture from computer work or looking down at a smartphone. As cervical vertebrae sublux anteriorly, a stretch compression can occur on internal jugular veins and autonomic nerves in the anterior neck, including the vagus nerves and cervical sympathetic ganglion [9,13].

Building on established associations in literature between (1) cervical spondylosis and excessive smartphone use in young patients, and (2) lifestyle and postural changes leading to visual symptoms, in which some cases the missing explanation could be LCI and cervical dysstructure causing carotid sheath compression [49,50]. To summarize these postural phenomena of using electronic devices, which lead to myriad symptoms, the term facedown/forward head lifestyle is used, which describes the many hours a day spent in an unnatural posture putting chronic low-grade forces on the spine while using electronic devices. FDFH posture causes slow stretching of the posterior ligament complex of the neck. Constant or repetitive stress on the ligaments over time will lead to ligament laxity, a process known as creep. The C1–C7 facet joint capsular ligaments are prone to injury and dysfunction as a result of excessive strain on the cervical spine from repetitive use in abnormal posture, which can result in LCI (Figure 2).

### 3.2. Facedown/Forward Head Lifestyle Leads to Ligamentous Cervical Instability

With an FDFH lifestyle, the lower segment of the cervical spine (C2–C7) may suffer first, as seen with the common forward head posture, and the upper cervical spine compensates for flexion of the lower cervical spine with hyperextension of the suboccipital region (C0–C2) in order to maintain horizontal gaze [51,52,53]. Pathologic capsuloligamentous laxity in the neck, especially the upper cervical region, causes LCI (upper and lower), leads to a breakdown of the cervical lordotic curve (cervical dysstructure), and the change in the upper cervical anatomy puts additional strain on the ligamentous support, which eventually fails, resulting in a forward-displaced atlas (C1) [54,55]. The forward shift of the atlas ultimately causes dynamic carotid sheath compression and, if left unresolved, eye and visual symptoms.

Primary consequences of LCI on the eye causing visual symptoms and diseases could be due to the net effects of the forward-shifted atlas, causing dynamic carotid sheath compression: obstruction of venous drainage of the eye and brain by compression of the internal jugular veins, and ocular dysautonomia in part from stretch and degeneration of the vagus nerves [10,56,57,58,59,60,61,62].

This hypothesis considers LCI as a catalyst for elevating intraocular pressure (IOP) and intracranial pressure (ICP), breaking down the blood–ocular barriers, causing “double-eye squish,” and ultimately resulting in a toxic, polluted eye ecosystem. Potential mechanisms affecting the eye and visual pathways following internal jugular vein (IJV) compression include interference with eye fluid flow hydrodynamics, eventual breakdown of the mechanical stability of the eye in the corneoscleral shell, liquefaction of the vitreous humor, and elevated cerebrospinal fluid pressure around the optic nerve head [63]. Mechanisms affecting the eye due to vagus nerve degeneration could include autonomic nervous system dysfunction, such as dysregulated blood flow, chronic pupil dilation and ophthalmic artery spasms.

## 4. Text Neck, Computer Vision Syndrome, Neck Pain, and Pathology

Not surprisingly, up to 90% of computer and cell phone users experience symptoms, and many patients are receiving diagnoses of computer vision syndrome (CVS) and text neck syndrome (TNS) [64,65]. While CVS emphasizes computer use and TNS relates to cell phones, FDFH encompasses both, and it is possible they mutually result from detrimental effects on the cervical spine: ligamentous cervical instability.

**Figure 3:**
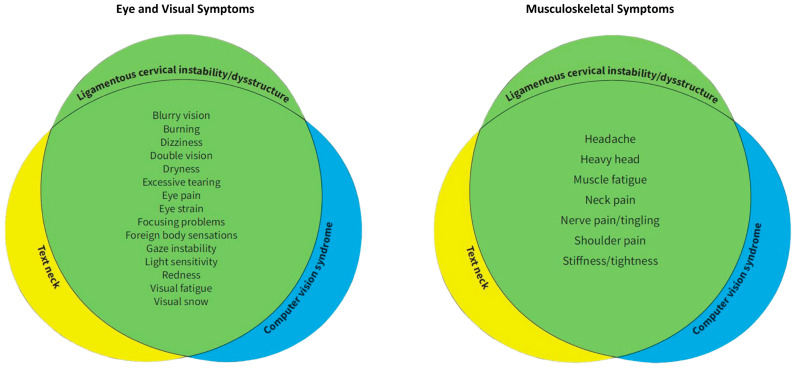
Venn diagrams demonstrating the commonality of eye, visual, and musculoskeletal symptoms in ligamentous cervical instability, text neck, and computer vision syndrome [9,64,65,66].

Text neck and CVS typically present with symptoms characterized as ocular symptoms, including eye pain and strain, dryness, irritation, and burning, and vision symptoms such as blurriness and double vision, as well as musculoskeletal symptoms, including neck, shoulder, and back pain [4,66]. CVS, TNS, and LCI share many similar eye, vision, and musculoskeletal symptoms, suggesting a common etiology between CVS and TNS (Figure 3). LCI, due to prolonged abnormal forces on the neck causing ligament creep, could be an underlying etiology of both CVS and TNS, the resultant visual symptoms being termed cervical oculopathy. Future studies investigating CVS and TNS should consider the cervical spine as a contributor. Similarly, studies investigating the link between eye symptoms and the cervical spine should consider lifestyle and facedown/forward head posture as a preceding factor.

It may be that some patients’ neck pain, cracking, and popping, and their eye and visual symptoms have a common pathology of LCI, but that the pathology could have stemmed from an FDFH lifestyle leading to LCI and causing carotid sheath compression, when no other cause is known.

A prior study by Hauser et al., 2025 evaluated a cohort of 203 consecutive patients who suffered with at least 1 of 6 eye symptoms (light sensitivity, blurry vision, eye pain/pressure, vision changes, seeing flashes of light, and eye tearing), aged 20–50 with no previous trauma or etiology for their chronic disabling neck, eye, and visual symptoms [9]. The data demonstrated that over 95% of the patient population had compression of the carotid sheath at the level of the atlas (C1), identified by a decrease in the cross-sectional areas of the internal jugular veins and vagus nerves, and significant ocular pathology was documented [9].

While much is known about the various risk factors for CVS and TNS, the underlying neurological pathology for the eye and visual symptoms is still poorly understood. Known risk factors for CVS and TNS include hours on the computer, duration of uninterrupted computer usage, ergonomic setup, proper lighting, computer glare, and blue light exposure, although some symptoms persist even with traditional treatment of known eye diseases, such as usage of eyeglasses [67,68]. Another clue that both the musculoskeletal and eye/visual symptoms of CVS stem from a common pathology is that CVS patients with neck pain experience visual symptoms at a higher rate than those without neck pain [65]. Time spent on devices generally correlates with the severity of musculoskeletal and visual symptoms [69]. Considering that the average person with CVS has multiple symptoms, including a range of ocular complaints and musculoskeletal pain, it is probable that the symptoms have a common origin [4]. Further, neck pain is commonly associated with visual symptoms, including reading and focusing issues, sensitivity to light, blurry vision, double vision, visual fatigue, visual objects moving, problems judging distances, red eyes, tearing, and others [70].

### Visual Symptoms in Ehlers-Danlos Syndrome and Whiplash Suggest Ligamentous Etiology

Some additional indications that a ligamentous etiology could explain both the ocular and muscular symptoms in some patients include the high prevalence of eye symptoms in hypermobile Ehlers-Danlos syndrome (hEDS) and post-whiplash injury, along with the fact that the female sex is an identified risk factor for CVS, as females are known to have weaker cervical spines [71,72,73]. Visual symptoms frequently identified in hEDS patients include dry eyes, blurry vision, diplopia, light sensitivity, and double vision, 70% being female [74]. A significantly greater amount of visual symptoms and ocular pathology was seen in patients with hypermobile EDS compared to a gender-matched control group, including lower tear film break-up time and Schirmer 1 test, lens opacities, pathologic myopia and vitreal abnormalities, steeper corneas, higher Best Fit Sphere index, and on confocal microscopy, lower density of cells in superficial epithelium and higher density of stromal cells’ keratocytes in anterior and posterior stroma [75]. EDS patients with upper cervical instability who underwent stabilization surgery experienced a significant reduction in both musculoskeletal (headache and neck pain) and visual symptoms (double vision, photosensitivity) [76,77].

In patients diagnosed with chronic whiplash-associated disorders or post-whiplash syndrome, approximately 70% complain of pain, dizziness, and balance issues, while 50% report eye and visual symptoms [78]. Up to 55% of those involved in whiplash injuries from car accidents have some significant symptoms 12–14 years later, including visual disturbances and a high prevalence of neck stiffness, though the reason for long-term symptoms remains largely unknown [79]. Building on the principle that “whiplash syndrome” is a central nervous system disorder, the nervous system dysfunction may be due to unresolved ligament injury resulting in ligament laxity, cervical instability, and nerve irritation [80].

Neck pain and visual complaints are also seen in occupations that require prolonged hours in a facedown posture similar to text neck, therefore putting excess strain on the posterior ligament complex of the cervical spine. In a group of 290 surgeons that perform minimally invasive surgeries, the prevalence and severity of neck/shoulder problems and visual symptoms were significantly associated (*p* < 0.0001) [81]. Another study on surgeons showed a positive relationship between the degree of visual impairments and muscle fatigue of the neck/shoulder muscles [82].

## 5. Cervical Lordotic Curve

The 7 vertebrae that make up the cervical spine are generally referred to in 2 distinct regions: the upper cervical spine (C0–C2), commonly referred to as the craniocervical junction (CCJ), and lower cervical spine (C3–C7) [83]. The lower cervical spine consists of intervertebral discs that are not present in the CCJ, making the lower cervical spine inherently more stable than the upper cervical spine [84]. The C1–C2 joint represents the most mobile segment in the vertebral column, accommodating the need to occasionally move the head extremely quickly and rotate left and right [85].

A lordotic cervical curve is the ideal C-shape of the cervical spine that spans from the foramen magnum to the thoracic spine, made-up of 7 precisely wedge-shaped vertebrae (C1–C7) that, when in optimal sagittal alignment (Cobb angle of 20–30°), maximize the spine’s ability to handle forces, balance and support the head, and maintain global spinal alignment [86,87]. The head is naturally positioned on the most mobile segments of the spine to allow for optimal function and range of motion, but C1–C2 is also the most vulnerable segment when the cervical spine undergoes increased forces such as in the FDFH lifestyle, as well as the most susceptible to injuries such as whiplash or trauma involving quick turning of the head [88]. Upholding cervical lordosis reduces forces on the structure of the neck by keeping the center mass of the cranium posterior to the lordotic curve [89]. FHP imposes approximately 3.6 times more pressure on the neck than a normal cervical lordotic curve [90]. In FHP, the center of gravity of the head is positioned anteriorly to the axis line through the shoulders, and the upper cervical spine (C0–C2) goes into extension while the lower cervical spine (C3–C7) goes into flexion [91]. With this altered anatomy, the depth of the cervical curve is decreased; a normal cervical lordosis depth is between 7–17 mm as evaluated by the Borden method [92,93]. Optimized function of the cervical spine and all of the neurovascular structures that run through it is achieved when it is in a lordotic state, with minimum tension present in the soft tissues (a relaxed state).

## 6. Documenting Cervical Structural and Dynamic Carotid Sheath Compression

**Figure 4:**
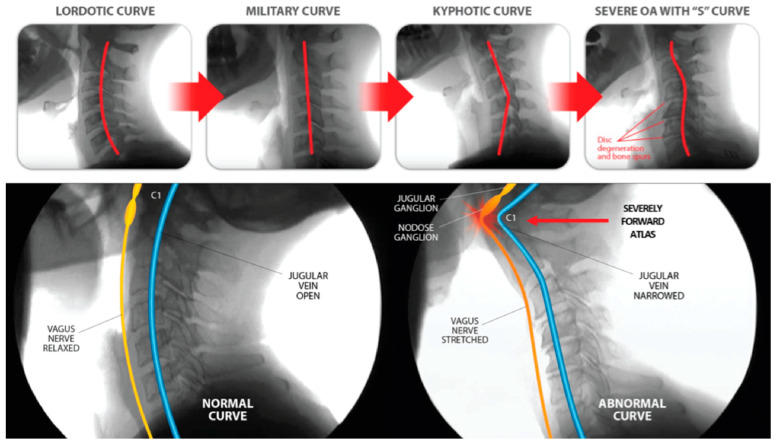
Cervical dysstructure degenerative progression and potential implications of internal jugular vein and vagus nerve compression.

The FDFH lifestyle causes a slow stretching of the posterior ligament complex of the neck, ultimately stretching and compressing the carotid sheath, causing stretch and compression on the IJVs and vagus nerves [94,95] (Figure 4). If the problematic forces continue, or the underlying ligament laxity is not dealt with, the structural dysfunctions will progress.

### 6.1. Cervical Structural Imaging

In-office dynamic cervical structural and carotid sheath testing, as well as noninvasive tests for intracranial hypertension, have been described previously [96]. These objective tests can be used to document not only the cervical structural abnormalities, but also the pathology it is causing. Periodic repeat testing after treatment has started can also document progress.

**Figure 5:**
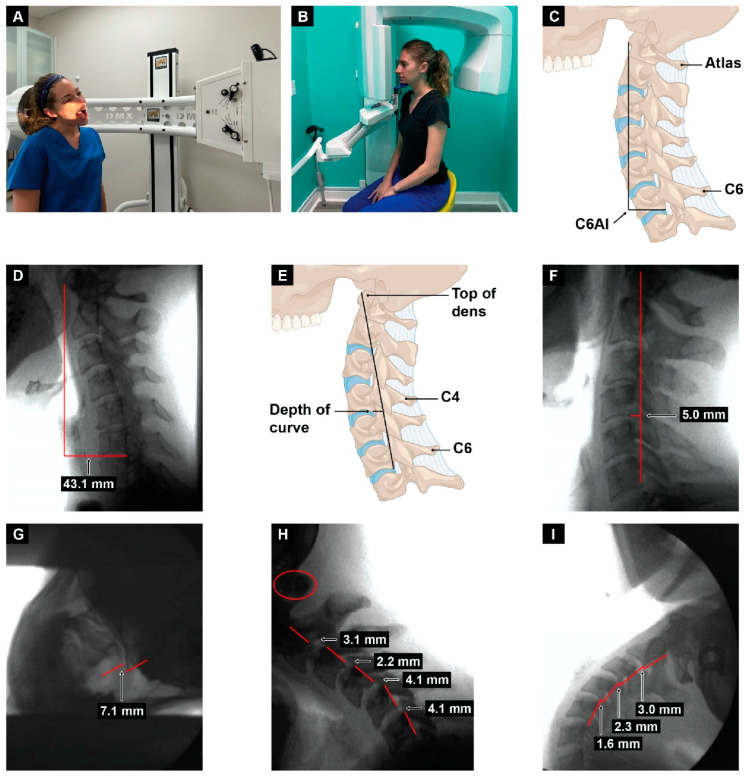
Upright digital motion (fluoroscopic) X-ray (DMX) and cone beam computed tomography (CBCT) scan with structural measurements. (**A**) DMX positioning for open mouth lateral flexion. (**B**) CBCT setup. (**C**) Forward head (C6AI *) illustration. (**D**) C6AI measurement. (**E**) Depth of curve ** illustration. (**F**) Depth of curve using DMX. (**G**) C1–C2 instability measurement. (**H**) Flexion, lower cervical instability. (**I**) Extension, lower cervical instability. * C6AI = horizontal distance in the sagittal plane of the posterior inferior C6 vertebra to anterior atlas (optimal is <10 mm) [9]. ** Depth of curve = horizontal distance in the sagittal plane from posterior inferior C4 vertebra to line drawn from posterior inferior C6 vertebra to top of dens (optimal is 7–17 mm) [92].

Cervical structural dysfunction is documented by digital motion (fluoroscopic) X-ray (DMX) and upright cone beam CT scan. LCI is assessed by continuous and detailed examination of cervical spinal movements in multiple dimensions, including the sagittal, rotational, and frontal planes. DMX is a type of motion videofluoroscopy and is a valid measure for cervical spine ligament injury [12]. The most common instability seen is ligamentous C1–C2 facet joint instability, which is found by doing an open-mouth view and the head placed in lateral flexion (Figure 5). Other co-structural pathologies are found with upright cone beam CT scan, including malocclusions, temporomandibular joint degeneration or instability, and elongated styloid bones (which can also compress the carotid sheath) [97,98].

The progressive nature of LCI, especially if the person continues to have an FDFH lifestyle, can eventually cause the cervical curve to reverse (become kyphotic) as the center of the mass of the cranium shifts further anteriorly, continuing to exert unnatural forces on the cervical spine, which can triple the forces on the posterior neck region, such as those seen when looking down at a cell phone, and is a catalyst for further cervical ligament injury or degeneration [99,100,101]. Since cervical ligaments and upper cervical muscles contain a high percentage of neck proprioceptors, it is known that injury to cervical ligaments causes an alteration in neck sensory input, which negatively impacts gaze and thus postural stability, causing gaze instability with various positions and postures of the head and neck through impairment in the cervico-ocular and vestibulo-ocular reflexes [102,103]. Disruptions to cervical sensory input can lead to many symptoms, including imbalance and dizziness with various trunk and head motions [104,105]. Injury to the cervical facet joint capsular ligaments is a potential cause for these effects [106]. Concurrently, the chronically flexed posture of the cervical spine compared to ideal cervical lordosis causes a stretching of the various neurovascular structures that run through the neck, including the spinal cord, sympathetic nerves, and those within the carotid sheath [107].

### 6.2. Dynamic Carotid Sheath Compression

Dynamic carotid sheath compression is a condition whereby changes in head and/or neck position, or motion cause a detrimental compression of the structures within the carotid sheath, hampering their optimal functioning. The structures most vulnerable to the stress and compression that occur through the cervical spine, especially at the atlas, are the internal jugular veins and vagus nerves. This dynamic compression, as well as the severity of it, is easily documented by positional cervical ultrasound examinations. Since the IJV is the most compressible component within the carotid sheath, IJV compression at the atlas can also be confirmed by CT venogram of the neck [108].

**Figure 6:**
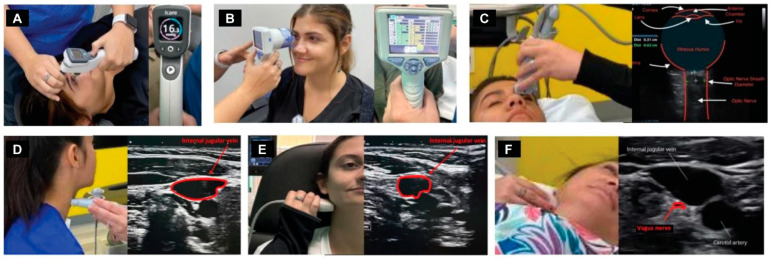
Neck vitals analysis. (**A**) Tonometry. (**B**) Pupillary light reflex. (**C**) Optic nerve sheath diameter. (**D**) Internal jugular vein (IJV) cross-sectional area (CSA) at C4–C5. (**E**) IJV CSA at C1. (**F**) Vagus nerve CSA.

Dynamic carotid sheath compression and the pathophysiology it causes, including intracranial hypertension, is documented by a testing procedure termed “neck vitals,” which includes tonometry, pupillometry, positional internal jugular vein cross-sectional areas, vagus nerve cross-sectional areas, orbital and cervical ultrasound with and without Doppler, and transcranial Doppler (TCD) examinations (Figure 6).

The vagus nerve can be easily seen with high-resolution ultrasound, as the most common location of the nerve in the mid-neck is lying posterior to the IJV and lateral to the carotid artery [109]. The vagus nerve cross-sectional area can then simply be measured, with normal cross-sectional areas being between 2–3 mm [110,111]. Studies have shown that the right vagus nerve tends to be larger than the left [112]. The optic and vagus nerves are the only cranial nerves easily and accurately measured noninvasively using ultrasound imaging, and both nerves can give us great insight into what may be causing eye disease.

**Figure 7:**
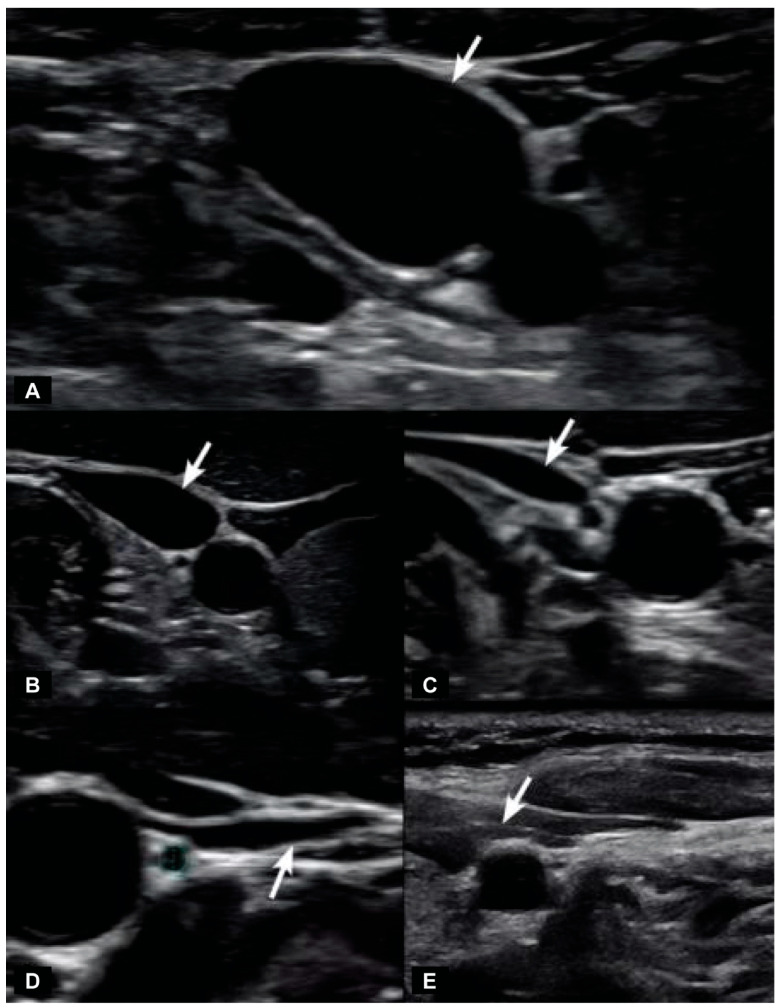
Degrees of internal jugular vein compression as seen on ultrasound examination of the neck. (**A**) Normal “open” internal jugular vein. (**B**) Slightly compressed. (**C**) Moderately compressed. (**D**) Severely compressed. (**E**) Completely closed. Internal jugular vein compression (arrows), especially with upright posture and neck motions, can lead to intracranial hypertension (increased brain pressure) in some cases [14,115].

**Figure 8:**
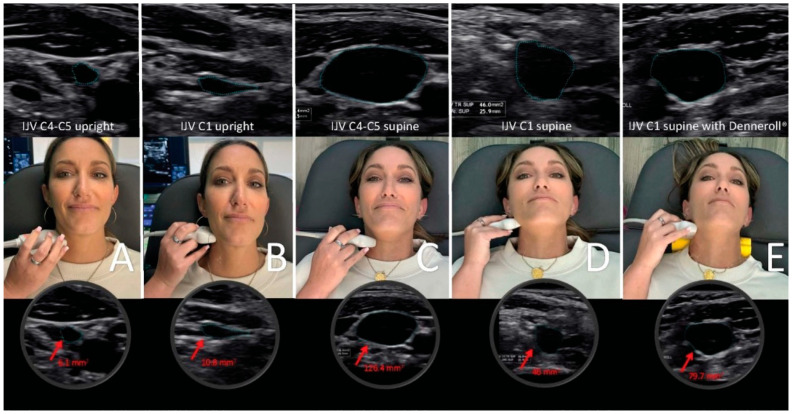
Internal jugular vein (IJV) cross-sectional area (CSA) measurement using ultrasound in various head and neck positions. (**A**) Upright measurement at the C4–C5 level. (**B**) Upright measurement at the atlas (C1) level. (**C**) Supine measurements at the C4–C5 level while lying on the Denneroll^®^. (**D**) Supine measurements at the C1 level. (**E**) Supine measurements at the C1 level while lying on the Denneroll^®^.

**Figure 9:**
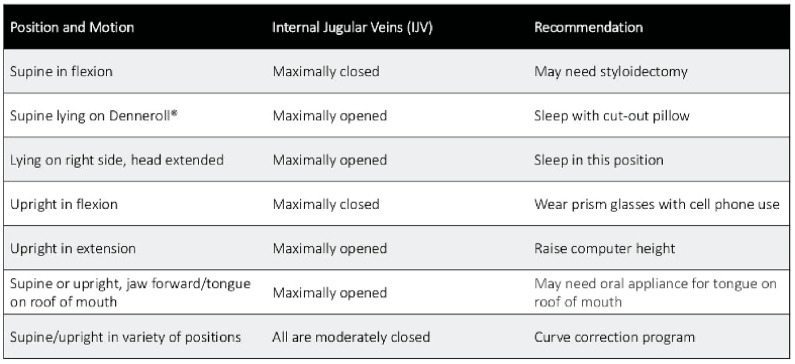
Potential scenarios of the internal jugular veins in various head, neck, and jaw positions, and possible recommendations to consider based on this information.

The jugular vein is also easily accessible by noninvasive ultrasound scanning [113]. Like all veins, the internal jugular vein does not have a muscular wall, so it is easily compressible (Figure 7). A normal IJV cross-sectional area in the supine position is above 90–100 mm^2^ regardless of head position [114,115,116]. Measuring the cross-sectional area of the IJVs in the mid-neck (C4–C5) and upper neck (C1–C2) using ultrasound in both a seated and supine position and with different head and neck positions can determine if they are being compressed and by how much (Figure 8). Knowing which positions cause maximum opening of the IJVs in the supine position helps in making many recommendations for future care, including best sleeping position, computer or tablet height, or whether there can be other factors involved in IJV compression, such as mandibular position or elongated styloid bones (Figure 9). The IJV compression is often at the level of the atlas (C1) and just checking the IJV at the C4–C5 level, which is the typical location, can miss critical IJV narrowing [117].

It is proposed that 2 primary mechanisms are at play in an apparently healthy younger population whose unresolved eye and visual symptoms are from LCI and CD due to carotid sheath compression: (1) impaired venolymphatic drainage of the eye and brain by compression of the internal jugular veins, and (2) ocular dysautonomia from a disruption to the parasympathetic/sympathetic system balance due in part to vagus nerve degeneration. These 2 mechanisms may be overlooked catalysts for ocular dysfunction by potentially causing changes in normal ocular fluid flow, hindering ocular automimic control, increasing eye and brain pressures, and elevating cerebrospinal fluid pressure and fluid around the optic nerve head.

## 7. Internal Jugular Vein Compression

Emerging evidence suggests that pathology associated with IJV compression emphasizes the importance of optimal venous outflow, especially from the brain, yet the factors leading to IJV compression are not yet fully understood [60]. IJV compression, or stenosis, and the increase in central venous pressure that results, is a known cause of intracranial pressure and has been speculated to be involved in the pathophysiology of myriad conditions or symptoms involving the eye, including transient monocular blindness, Ménière’s disease, multiple sclerosis, Alzheimer’s, and idiopathic visual changes such as visual loss, blurred vision, diplopia, dry eye, eye pain, and some non-ophthalmological, such as sixth cranial nerve palsy, vertigo, head pressure, presyncope, and photophobia [118,119,120,121].

The primary cerebral venous drainage on 94% of people is the IJVs, and then also through the lymphatics, deep cervical veins, and extrajugular vessels while in the upright position and if the IJVs are blocked due to LCI (for example, if the atlas is unstable and shifting forward, causing compression) [122,123,124] In a study of over 145 patients with IJV compression, 91.2% of subjects had radiological findings of jugular vein stenosis at the J3 (upper) segment, 28.9% of subjects reported visual impairment, 36.2% had increased intracranial pressure, and 12.9% presented with papilledema [125]. IJV compression is an increasingly recognized cause of elevated ICP, and occurs at the level of the transverse process of C1 and styloid process (J3 segment of IJV) in up to 96% of cases, supporting the notion that an under-researched cause of IJV compression is ligamentous cervical instability causing a forward shift of C1 (atlas) [126,127,128]. Compression of the IJV, especially right below the foramen at the level of C1, impairs hemodynamics of the brain, which can result in sudden or chronic increase in cerebral blood volume (intracranial pressure), altering cerebral venous and CSF dynamics, both potentially leading to elevated IOP [129]. The upper segment of the IJV (J3) is the most susceptible to being compressed by upper cervical pathology, but other causes of IJV compression include mandibular malposition, hyoid bone, sternocleidomastoid muscles, and elongated styloid bones, among others [130,131,132]. These positions also have the potential to cause vagus nerve dysfunction, as the vagus nerve runs alongside the IJV in the carotid sheath.

Veins are easily compressible since they have no muscular wall layer for protection. Simple neckties and cervical immobilization devices have been shown to compress the IJVs [133,134]. The ease with which the IJVs can be compressed and the significance of this fact show that mean jugular venous pressure, IOP and ICP (normally 10–15 mmHg) can increase 40–70%, 7 mmHg and 5 mmHg, respectively, simply by wearing a cervical collar [135,136,137,138]. A cervical collar or necktie can also significantly increase optic nerve sheath diameters in just 5 min, as well as elevate cerebrospinal fluid pressure [139,140,141].

Essentially, IJV compression causes a fluid backup, like a kink in a hose. If the brain, and thus the eye, cannot drain properly, cerebrospinal fluid and venous pressures increase, and with it their ability to remove toxic metabolic waste products becomes hampered [142]. This build-up of fluid can increase pressure all the way back to the episcleral veins, which is where outflow from the orbits of the eyes (blood and aqueous humor) travels through before descending through the superior ophthalmic veins, which lead to the cavernous and petrosal sinuses, and exit the head through the internal jugular veins [143,144].

### Intracranial Pressure and Intraocular Pressure Affected by IJV Compression

**Figure 10:**
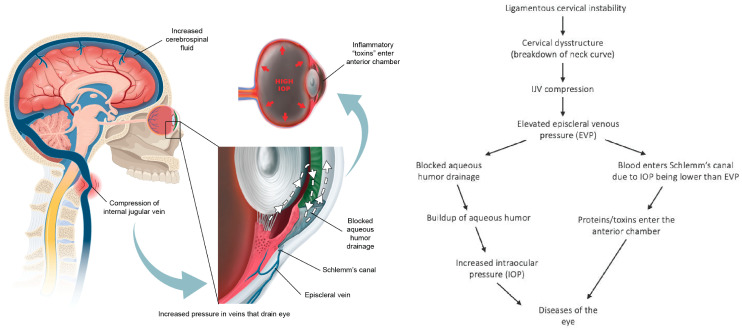
A potential ligamentous cervical instability etiology of eye diseases [145,147].

As ocular venous pressures increase, fluid flow within the eye itself becomes affected. In the regulation of IOP, various factors come into play, including the production of aqueous humor, resistance to its drainage at the anterior chamber angles, and episcleral venous pressure (EVP) [145]. An essential aspect of maintaining normal composition of aqueous humor is uninterrupted, unidirectional flow from the posterior chamber (where it is produced from the ciliary body) through the trabecular meshwork (sclerocorneal angle), and across Schlemm’s canal, where it exits the eye into the aqueous and episcleral veins, and then through the cavernous sinuses and eventually the IJVs [146,147]. *When EVP becomes higher than the IOP, ocular fluid flow can reverse, resulting in blood entering Schlemm’s canal, penetrating the endothelium, an important part of the blood–aqueous barrier, and plasma proteins crossing through to the anterior chamber (causing inflammation and creating a “toxic eye”)* [148] (Figure 10).

According to the Goldman equation, EVP accounts for approximately 47–60% of IOP in humans, and IOP is dependent on the EVP, as any rise in EVP will increase the resistance to aqueous humor outflow. This assertion is supported by studies that attribute a rise in IOP to elevated EVP when comparing postural pressure ocular changes [149]. Aqueous humor also has an unconventional outflow pathway, the uveoscleral pathway, accounting for only up to 35% of aqueous humor drainage, and thought to be independent of IOP [150]. Changes in ocular fluid hemodynamics could make the eye susceptible to numerous different degenerative conditions, including macular degeneration, retinopathy, cataracts, and glaucoma, although a substantial amount of research is required to fully understand if and when cervical spine structure is involved in this potential phenomenon [151].

When IJV compression leads to elevated intracranial venous pressures, the retinal vein and orbital pressures may also rise in an attempt to counterbalance and maintain the translaminar pressure gradient [148]. Typically, the EVP maintains stability within a range of 8–10 mmHg, with a correlation of increased EVP and elevation of IOP believed to be 1 mmHg:1 mmHg [152]. Normal values for central venous pressure, measured by Doppler ultrasound of the IJV, also typically fall within the range of 8–12 mmHg [153]. It is postulated that IJV pressure could be a predictor of elevated episcleral venous pressure due to the fact that IJV compression is known to cause increased intracranial pressure, and ICP is shown to produce upstream pressure changes, specifically related to EVP [153]. Notably, central venous pressures, akin to episcleral venous pressures, can be measured noninvasively using Doppler ultrasound on the IJV [154].

While there are studies that debate whether a correlation between IOP and ICP exists, many studies show a clear and consistent link, as well as suggest tonometry to be utilized to detect intracranial hypertension in a clinical setting [155,156]. The reason for discrepancies regarding this correlation could be that the underlying cause of altered IOP or ICP is often chronic though intermittent IJV compression because of LCI. The degree and frequency of compression would be dependent on head and neck positions, as well the degree of LCI and cervical dysstructure.

If there is vascular compression (IJV insufficiency), then it is conceivable that IOP can be an accurate predictor of ICP, making tonometry a potentially great noninvasive tool to detect increased ICP when there is LCI etiology. In such cases, restoring proper cervical structure and fluid flow through the IJV would resolve ICP and elevated IOP, and decrease accompanying visual symptoms associated with these conditions. Future prospective studies will be needed to establish this relationship.

## 8. Cerebrospinal Fluid Disruption Evidenced by Elevated Optic Nerve Sheath Diameter

The physiological importance of CSF flow includes its role in clearing brain and eye metabolic waste products through the glymphatic system [157]. The average human body is circulating approximately 150 mL of CSF at any given time, with the majority surrounding the central nervous system through the cisterns and subarachnoid spaces (SASs), including that which encompasses the optic nerve [158,159]. LCI and CD can directly increase CSF pressure by causing obstruction of CSF flow in the neck, or indirectly through compression of the IJVs. Anterior subluxation of the atlas, axis, and lower cervical vertebrae ranging from 3–6 mm can reduce axial spinal canal area by up to 60% [160].

Visual disturbances can arise due to increased CSF pressure and fluid building up around the optic nerve, which can be objectively measured as an increase in ONSD [161]. Increased CSF pressure on the optic nerve is responsible for myriad changes seen in the optic nerve with a variety of eye diseases, including optic neuritis and atrophy, as well as macular degeneration [57,162,163]. Elevated CSF pressure and abnormal CSF clearance are also associated with glaucoma development, identifiable by progressive damage to retinal ganglion cells [164,165]. Lowering CSF pressure has a direct and immediate effect on the optic nerves, as demonstrated on MRI [166]. The elevated CSF is responsible for MRI findings in intracranial hypertension such as posterior globe flattening, optic disc displacement, increased ONSD, tortuosity of the optic nerves, and flattening of the sclera [167,168].

## 9. Noninvasive Testing for Elevated Intracranial Pressure

**Figure 11:**
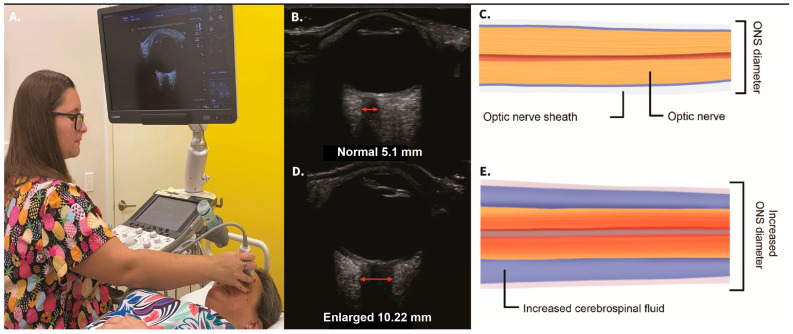
Ocular ultrasound demonstrating a normal and an enlarged optic nerve sheath diameter (ONSD). (**A**) Technique. (**B**) Normal ultrasound image. (**C**) Graphic pictorial of normal ONSD. (**D**) High ONSD image. (**E**) Illustration of extra cerebrospinal fluid around the optic nerve. A high ONSD is a noninvasive test for intracranial hypertension (brain pressure > 20 mmHg) [173,174].

When the IJV is occluded, leading to cerebral venous insufficiency-induced elevated ICP can be reflected as increased ONSD. The SAS of the optic nerve is a particularly vulnerable place for increased CSF pressure to build up, with outcomes such as papilledema, optic neuritis, and optic nerve tortuosity [169,170]. Elevated CSF pressure surrounding the optic nerve can be noninvasively documented by measuring the optic nerve sheath diameters using ocular ultrasound 3 mm from the posterior aspect of the globe, which is known to be the area most distensible and sensitive to elevated pressures [171,172]. Normal optic nerve sheath measurements are usually <5.7 mm in adults when the eye and brain pressures are normal (10–15 mmHg), whereas ONSDs > 6.1 indicate pathological brain pressures above 20 mmHg [173,174] (Figure 11).

Idiopathic intracranial hypertension produces increased pressure around the distal optic nerve, causing stasis of axoplasmic flow and eventually ischemia and a breakdown of the blood–retinal and optic nerve-blood barriers, progressing to bilateral blindness in 10% of patients [175,176,177]. Removal of CSF via lumbar puncture to decrease brain pressure in patients with idiopathic intracranial hypertension has an immediate effect on the amount of CSF in the optic nerve sheath, documented by decreased optic nerve sheath diameter before and after lumbar puncture [178].

Noninvasive testing for intracranial hypertension (ICH) includes ocular ultrasound for optic nerve sheath diameters, transcranial Doppler examination of middle cerebral artery velocities with pulsatile index, and tonometry for intraocular pressures [179]. Normal pressure within the lumbar and brain CSF is noted to be 6–15 mmHg in the supine and lateral decubitus positions, and slightly lower while upright because of gravity [180]. ICP monitoring using invasive methods (lumbar, brain, or cervical spine puncture) has been the gold standard for the evaluation of ICH, defined as a cranial pressure of greater than 20 mmHg, but limitations include its invasiveness and potential complications, such as hemorrhage and infection [181]. For this reason, noninvasive methods have been developed to document ICH (ICP > 20 mmHg), some of the more commonly used being ONSD, intraocular pressure, middle cerebral artery velocities, and pulsatile index [182,183].

The brain’s dura mater and subarachnoid space (which contains the CSF) are continuous with the posterior of the eye. The dural, pia, and arachnoid sheaths of the optic nerve are continuous with the sclera of the eye [184]. Changes in the CSF pressure in the brain can therefore be reflected around the optic nerve.

The optic nerve emerges from the posterior part of the globe and appears as a hypoechoic linear structure with a hyperechoic border (nerve sheath). The outer rim should be included in optic nerve sheath measurements and measured 3 mm behind the posterior rim of the globe (where standard measurements are made). Measurements in adults and children without ICP are typically around 3 mm +/− 1 mm [185]. While some studies in adults use a cutoff as low as 4.5 mm and as high as 6 mm as evidence for increased ICP, emergency rooms and most others use an ONSD measurement of greater than 5.5–6 mm to diagnose ICH [186,187,188,189,190].

Transcranial Doppler ultrasound provides real-time measurements of blood flow in the arteries that go to the brain, whereas extracranial Doppler examines the veins and arteries in the neck as they go into and out of the brain. TCD has been called the stethoscope for the brain and extracranial Doppler the stethoscope for the neck. These instruments can track moment-to-moment changes in blood flow to the brain from the vessels in the neck and can assess the effect of interventions such as changes in neck position on brain blood flow [191,192,193]. TCD can also document abnormalities in arterial velocities and pulsatile index, which can be noninvasive indicators of intracranial hypertension [194]. Generally speaking, a peak middle cerebral artery velocity (biggest end-branch of carotid artery) greater than 90 cm/second is abnormal [195]. The patient can be placed in head and neck postures and positions with the middle cerebral artery velocities and pulsatile index checked to see if they go up when IJV compression occurs. Pulsatile index, the difference between the peak and minimum diastolic flow velocities divided by the mean velocity, recorded throughout the cardiac cycle, reflects ICP levels from 5–40 mmHg, as well as cerebral perfusion pressures [196]. An easier way to think about it is that it is vascular resistance to pulsating, so if the middle cerebral artery in the brain has a high pulsatile index on one side, it likely means the pressure on that side of the head is much greater than the other side. Pulsatile indices greater than 0.92 are indicative of ICP greater than 20 mmHg [197]. If the IJV on that side of the neck is compressed, then that is the likely cause of the raised ICP on that side.

Tonometry, or IOP, is also part of the neck vital analysis, as ICH has many effects on the eye, including posterior globe flattening, modulation of aqueous humor dynamics, and elevations of eye pressure [198,199]. Abnormal IOP as measured with a handheld tonometer correlates with abnormal intracranial pressures [200,201]. ICH is a known risk factor for glaucoma (elevated eye pressure) [202]. It is easy to document the relationship between the jugular veins and IOP, as wearing a necktie can increase pressure by 2–4 mmHg and by doing a jugular vein compression test, it’s been shown that compression doubles IOP in real time [134,203,204].

## 10. Is Increased ONSD a Cause of Blurry Vision and Optic Nerve Dysfunction and Degeneration?

**Figure 12:**
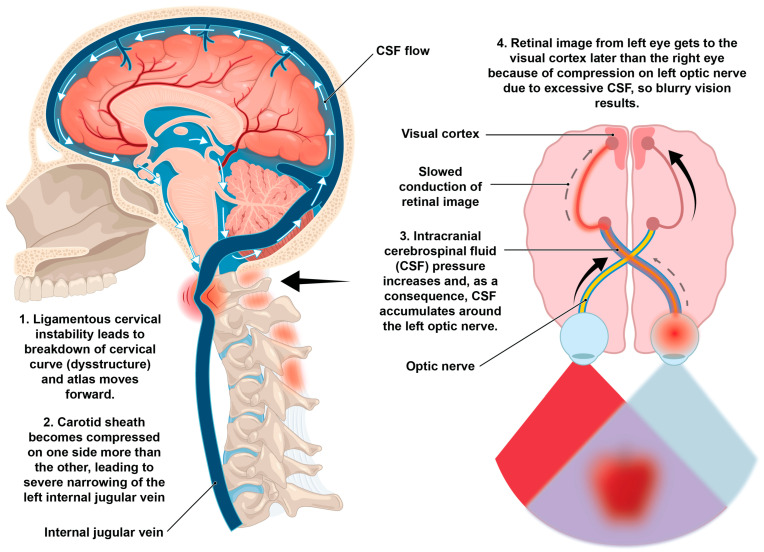
Potential ligamentous cervical instability etiology of blurry vision.

The optic nerve is the electrical cable system that brings electrical impulses from the retina to the brain at speeds of 3–13 m per second so we can see [205,206]. When cerebrospinal fluid accumulates within the optic nerve sheath, significant pressure can accumulate within the sheath, potentially limiting these impulses, and if left untreated, could cause the loss of optic nerve cells [165]. In conditions where CSF pressures are high, such as idiopathic intracranial hypertension, the increased pressure can impair axoplasmic flow within the optic nerve, causing a decrease in conduction velocity in the optic nerve due to compression of the nerve fibers within the optic nerve sheath [207]. A proposed possibility included in this hypothesis is that if the electrical signal from one retina to the occipital lobe goes slower than the other side because of CSF accumulation and pressure, blurry, double, or even afterimages can potentially occur (Figure 12).

### Optic Nerve Head Mechanical Stresses

The optic nerve head (ONH) is subject to mechanical stress by 2 primary forces: the pressure generated in the connective tissues circumferentially surrounding it in the peripapillary sclera from IOP, and stress produced by the translaminar gradient between the normally higher IOP and the lower optic nerve tissue pressure [208]. A key element of vision is normal pressure and fluid flow through the retina and optic nerve. The nerve fibers forming the optic nerve exit the eye posteriorly through a hole in the sclera that is occupied by the lamina cribrosa, a mesh-like structure in the optic nerve head that allows retinal ganglion cell axons to pass through to the brain [209]. The translaminar pressure gradient refers to 2 forces at the lamina cribrosa of the optic nerve: the anterior-acting ICP and the posterior-acting IOP.

IOP and ICP might be thought of as relatively independent pressure systems, but given that they both work to manage the translaminar pressure difference (IOP − ICP = TLPD) [210], and they maintain stability through appropriate circulation of aqueous humor and CSF, along with their respective neuroregulatory mechanisms, they are indeed interrelated [198]. The intraocular component of the TLPD is represented by IOP and the retrobulbar component is the orbital CSF pressure, which is directly related to brain CSF pressure, which directly relates to whether the IJVs are open (sufficiently draining) or not [211].

**Figure 13:**
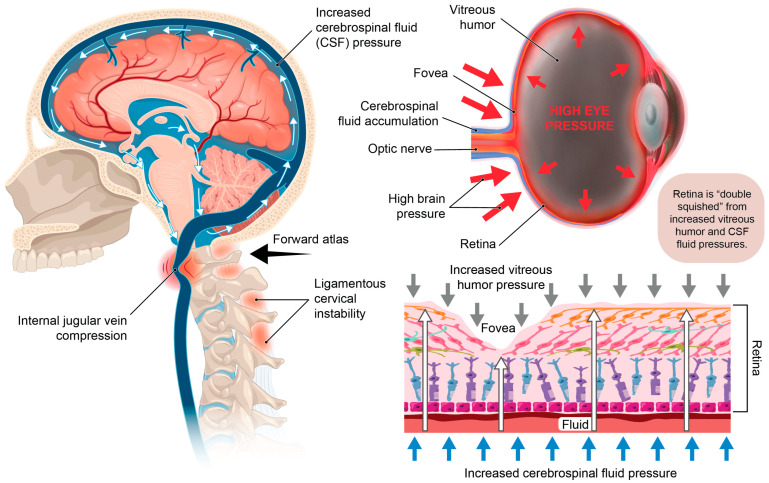
Potential structural pressures on the retina due to internal jugular vein compression. When the brain pressure increases from narrowing of the internal jugular vein, the posterior globe of the eye may get compressed, cerebrospinal fluid can accumulate around the optic nerve, and there can be a decrease in fluid flow out of the eye. The net effect may be elevation of intraocular pressure or ocular hypertension. The retina is subject to “double squish” between the high pressure on the outside of the eye and the elevated pressure within it, potentially leading to symptoms and diseases including blurry vision, double vision, focusing issues, glaucoma, and macular and retinal degeneration.

IJV outflow insufficiency interrupts the fluid flow dynamics of aqueous humor and CSF circulation, therefore affecting both IOP and ICP and dysregulating visual system pressures from inside and outside the globe, particularly causing problems at the optic nerve head where the 2 pressures collide [212] (Figure 13). The ONH is a unique structure of the eye that includes a blood–brain barrier, the blood–optic nerve barrier [213]. The optic nerve and cells are comprised of 12 million axons, the retinal ganglion cells, which are particularly vulnerable to neurodegeneration by excessive forces, even if intermittent [214].

There is a circumpapillary ring of elastin and collagen fibers in the immediate peripapillary sclera, providing structural support to the lamina cribrosa against elevated IOP, but the lamina is much thinner and weaker than the entirety of the sclera, making it subject to posterior displacement [215]. To maintain a healthy eye, the lamina and sclera must work together to regulate the constant pressure fluctuations of IOP and CSF and resultant strains on the ONH [208]. Optimal function of the retina greatly depends on the shape and stiffness of these structures. Increased brain or eye pressure has significant effects on the physical structure of the eye, specifically the optic nerve head, including deformations of the anterior lamina cribrosa and scleral canal at Bruch’s membrane opening, and both pressures can be altered due to IJV compression [216]. An additional mechanism may involve IOP rising due to elevated central venous pressures because of compression at the IJV backing up through the cavernous sinus toward the episcleral veins. An increase in CSF pressure leads to a corresponding rise in pressure within the optic nerve sheath, transmitting this increased pressure to the optic nerve and nerve head [211].

TLPD is typically low, around 2–4 mmHg; when it is increased, axonal damage to the optic nerve occurs, ref. [217] which means that IOP is normally slightly higher than ICP, causing a small, posteriorly directed pressure gradient across the optic nerve head. Abnormally high or low pressure on either side of the lamina cribrosa is an important consideration for ocular disease, meaning increased CSF pressure at the back of the eye could cause just as much damage as increased IOP is known to cause, and both can be elevated due to IJV compression, independently or concurrently [218]. When elevated, the 2 pressures may not “cancel each other out,” and it is permissible that they would cause increased damage to the posterior structures of the eye [216]. Thinning of the sclera and retina, for example, could be caused by elevated IOP increasing the diameter of the globe, or by degeneration of collagen due to destructive abnormal forces, as thinning of these structures is associated with retinal pigment epithelium cell death in myopic eyes [219]. Increased CSF in the optic nerve SAS, as well as impaired glymphatic clearance of waste products (leading to a toxic eye), both due to IJV obstruction, could lead to degenerative conditions such optic neuritis and glaucoma [220].

The optic nerve is exposed to IOP at the intraocular portions of the retina, choroid, and sclera, then extends through the lamina cribrosa, where it becomes surrounded by CSF [202]. CSF flow and pressure can be altered due to ligamentous cervical instability, inducing long-term abnormal pressures in the SAS of the optic nerve and ongoing pressure on eye structures at the back of the eye, such as the retina and sclera (especially affecting the lamina cribrosa), as well as the bulbous part of the optic nerve [221]. It has been proposed that the retina is exposed to CSF through the paravascular space of the retinal vessels, while the sclera takes on full-force CSF pressure at the optic nerve head, as the optic nerve head and brain share the same CSF pool [222]. An imbalance of the pressures acting on the lamina cribrosa may also result in a decrease in overall circulation of the fluid, meaning decreased inflow of fresh fluid and nutrients.

The ONH and lamina cribrosa are important not just for managing pressure and allowing retinal ganglion cells to pass through for formation of the optic nerve, but also for keeping vitreous waste out of the central nervous system (CNS) [223]. If the optic nerve head serves as a barrier between vitreous and the optic nerve, then damage/change to the optic nerve head (or damage to the lamina cribrosa) could allow infiltration by inflammatory vitreous into the optic nerve, such as when the aqueous humor is blocked, IOP is building, and waste is accumulating. It is possible that a breakdown in this barrier could also allow infiltration of CSF, when CSF does not normally cross this barrier. In glaucomatous eyes, however, it is not yet shown that this transfer happens.

Lowering CSF pressure has a direct and immediate effect on the optic nerve, demonstrated by MRI findings revealing decreased optic nerve tortuosity directly following removal of CSF, and further concluding that optic nerve tortuosity and optic nerve sheath diameter measurements are useful in determining ICP changes [166]. Correcting the structural cause of high CSF pressure when applicable would be the most ideal and beneficial solution for protecting, and possibly reversing damage to, the optic nerve and lamina cribrosa. An important consideration is restoration of the lordotic cervical curve and Prolotherapy treatment to resolve LCI, thereby opening the CSF drainage pathways to return to normal CSF pressure.

## 11. Leaky Eye: Disruption of Blood–Ocular Barriers and Increased Pressures from Internal Jugular Vein Compression

Like all organs of the body, the eye has nutrients constantly flowing into it and waste products out of it. This flux keeps the 2 main fluids—the vitreous body and aqueous humor—clear and healthy to maintain the eye’s shape and allow light to penetrate freely through the pupil to the retina. The eye actually has its own normal ocular biomechanics that are dependent in large measure on the fluid flow in and around the eye, as the posterior chamber gel-like vitreous humor maintains the eye’s spherical shape and buffers it from mechanical stimuli exerted on the lens, retina, and other eye structures under both static and dynamic influences due to its viscoelastic properties [224,225]. Vitreous humor is a transparent hydrogel situated between the lens and the retina and is the largest component of the eye, occupying 80% of the eye’s volume. Many eye conditions, and even aging, are characterized by vitreous liquification, which compromises its shock-absorbing capacity, leading to or complicating conditions such as retinal detachment, vitreous floaters, macular holes, vitreous hemorrhage, glaucoma, macular degeneration, and retinopathy [226].

Postmortem vitreous humor levels of protein biomarkers such as total tau and Amyloid-ß were significantly increased in patients with Alzheimer’s disease and chronic traumatic encephalopathy compared to controls [227]. In one study, all 6 biomarkers for Alzheimer’s disease, such as Amyloid-ß, tau protein, glial fibrillary acidic protein, and neurofilament light chain, were found in greater quantitates in eye fluids (aqueous humor, vitreous humor, and tears) than in plasma [228].

It is further hypothesized that the discussed mechanisms initiated by LCI can also potentially disrupt waste product removal out of the eye and lead to disruption of the blood–ocular barriers [229,230]. This hypothesis is formulated on the basis that fluid flow out of the eye is disrupted, a breakdown in the blood–ocular barriers can occur, and the accumulation of inflammatory toxic metabolites within the eye fluids and the eye structures themselves, such as the retina, can occur. The 2 main blood–ocular barriers are the blood–aqueous barrier and the blood–retinal barrier (BRB). Less commonly considered, but also important to pay attention to as previously discussed, is the blood–optic nerve barrier.

The retina features a clearly defined anatomical foundation, as evidenced by the presence of specialized intercellular junctions in both the endothelium and epithelial cells bordering capillaries [231]. The BRB plays a crucial role in the eye by regulating the passage of molecules between the bloodstream and the retina, acting as a gatekeeper for circulating toxins. Its integrity is essential for maintaining a precisely controlled microenvironment, which ensures optimal mechanical properties of the retina [232]. Without the BRB, the retina would be susceptible to any rapid fluid flow changes in the eye.

**Figure 14:**
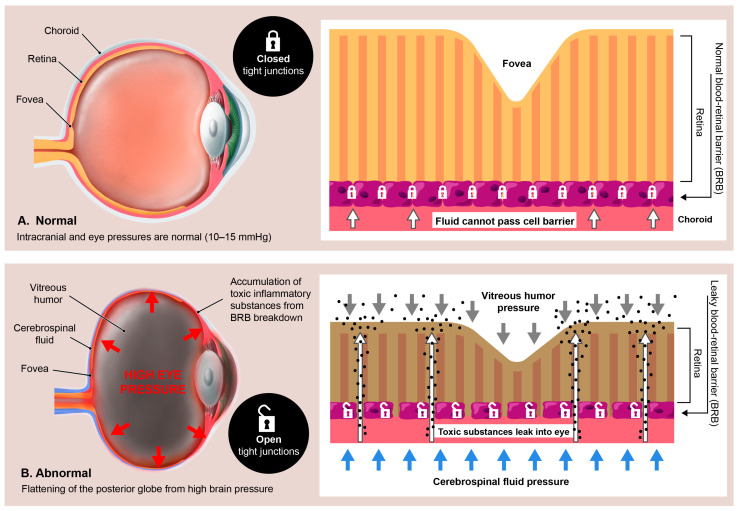
Hypothetical relationship between the brain/eye pressures and blood–retinal barrier. When the brain and eye pressures are normal, the barrier between the blood and the retina is tightly closed, but when the brain and eye pressures are elevated, the “tight” junctions become leaky, and toxic/inflammatory substances can then seep into the eye.

Disruption of the blood–retinal barrier is a common pathogenic pathway in many visual diseases that are otherwise considered to have exclusive characteristics, including macular edema (swelling of the macula), macular degeneration, and primary open-angle glaucoma [233,234]. Damage to the BRB involves loss of barrier cells and leaky blood vessels, which allow for inflammatory mediators, proteins, and other solutes that normally stay in the blood to reach the vitreous humor and retinal tissues, leading to macular edema [235] (Figure 14). This damage is due to a breakdown in the normal tight junctions, allowing for permeation of the retinal pigmented epithelium or retinal capillaries [236]. It is acknowledged that trauma, infections, diabetes, and hypertension are all known to cause direct damage to the BRB, but so can conditions that disrupt venous flow, such as central retinal vein occlusion [237,238].

**Figure 15:**
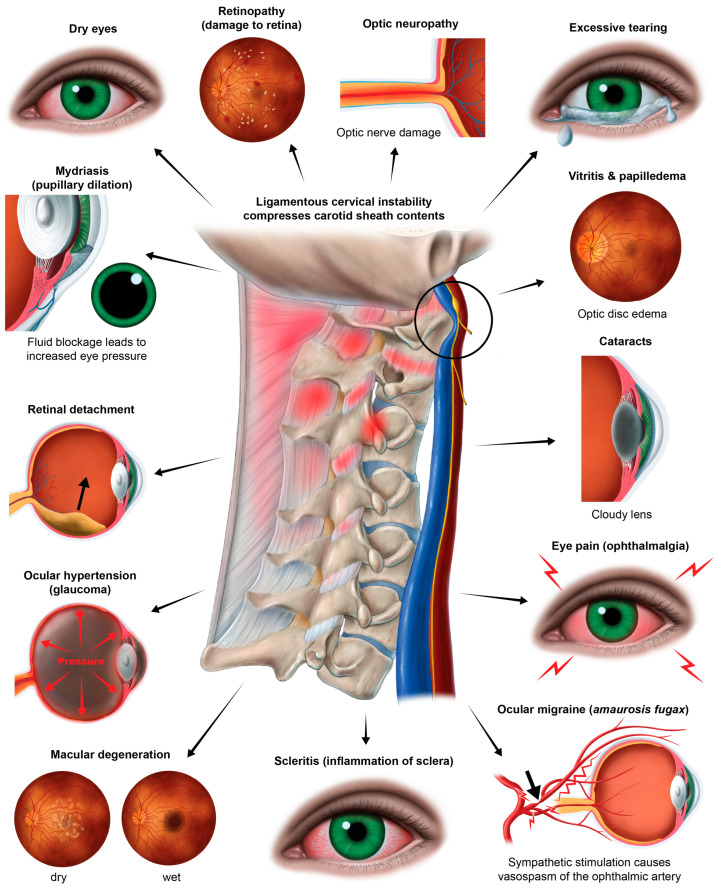
The many eye conditions potentially caused by ligamentous cervical instability and cervical dysstructure.

It is generally accepted that the proximate cause of macular edema and retinal fluid accumulation with many eye conditions and diseases is a breakdown of the BRB [239]. The retinal blood supply exits the eye at the optic discs via the central retinal veins and typically reaches the internal jugular veins via a route through the cavernous sinus [240,241]. A compressed jugular vein is like a traffic jam down the road: nothing can get out of the eye and brain, and everything gets backed up. Not only can the buildup of fluids cause pressure that results in inflammation, but the toxins that are supposed to be removed via the venous system will build up, too. It is highly likely that the chain of events which potentially follows a compressed jugular vein includes altered fluid flow dynamics of the venous system of the eye, which could negatively impact ocular fluid hydrodynamics, but this connection remains undetermined. Many potential eye conditions may arise from the effects of LCI and CD, some due to the eventual breakdown of the BRB and “toxic” eye (Figure 15).

The same structural neck issues and pathology that could allow inflammatory substances to accumulate in the vitreous humor can also do so in the aqueous humor. The aqueous humor is a transparent fluid produced by the ciliary body into the area behind the iris, where the ligaments of the lens and the ciliary muscle connect to the eyeball at a rate of 2–3 microliters/minute, flowing from the posterior to anterior chamber of the eye, serving the dual purpose of supplying nutrients for the cornea and lens and eliminating waste products [242]. Aqueous humor fluid flow has been known to be inhibited by elevated intraocular pressure, pupillary dilation, and elevations of EVP which, as part of this hypothesis, are suspected to have underlying IJV compression in some cases [226,243].

The ideas presented here are theoretical, intended as a conceptual expansion of the primary hypothesis, and provide a basis for further empirical explorations.

## 12. The “Double Eye Squish”

Altered fluid flow is one of the most well-known causes of visual impairments, and a known contributor of glaucoma, a leading cause of blindness [244]. Glaucoma is characterized by damage to the optic nerve and retinol nerve fibers, with suspected etiologies of elevated IOP, neuroinflammation, and recently, multiple abnormal pressures and central visual pathway dysfunction [245,246]. This neurodegenerative disease includes the optic nerve head and is associated with toxic inflammation, leading to cell death and disease progression. Keeping in mind there are many presentations of glaucoma, it is important to note there are still unidentified etiologies.

Aqueous humor drainage of the eye and CSF drainage of the brain are quite comparable in complexity, as both fluids nourish and remove toxins from respective tissues and structures of the brain and eye. The 2 fluids circulate with a similar dynamic and run their course through a dynamic pressure system which, when altered, can trigger a diseased eye state [242,247]. As a repercussion of the “kinking” of the venous hoses (IJVs) that drain both the brain and eyes, pressure across the retinal layers could increase both within the eye globe and outside the eye, causing a “double eye squish” in some cases.

Another important structure that provides stable mechanical support to internal ocular structures such as the retina and optic nerve head is the sclera, a part of the corneoscleral shell [248]. The sclera forms 85% of the outer lining or sleeve and is composed of connective tissue collagen that biomechanically helps “stabilize” the retina, optic nerve head, and lens-iris diaphragm. The sclera must do so under dynamic loading conditions imposed by eye movements and various ocular fluid flow pressures. Like other tissues, the collagen fibers of the sclera have a natural waviness known as “crimp,” giving the collagen its native dynamic strength with the ability to stretch if needed under dynamic load. Healthy scleral extracellular matrix is tremendously resilient to compression (nearly incompressible) [249]. If the chronic pressure generated from conditions such as ocular hypertension and glaucoma are not stopped by the sclera, pressure-induced stress will ultimately result in the death of retinal ganglion cell axons as the sclera becomes thinner, causing the corneoscleral shell to lose its viscoelastic properties and allowing higher pressures onto the retina and optic nerve head [250,251].

**Figure 16:**
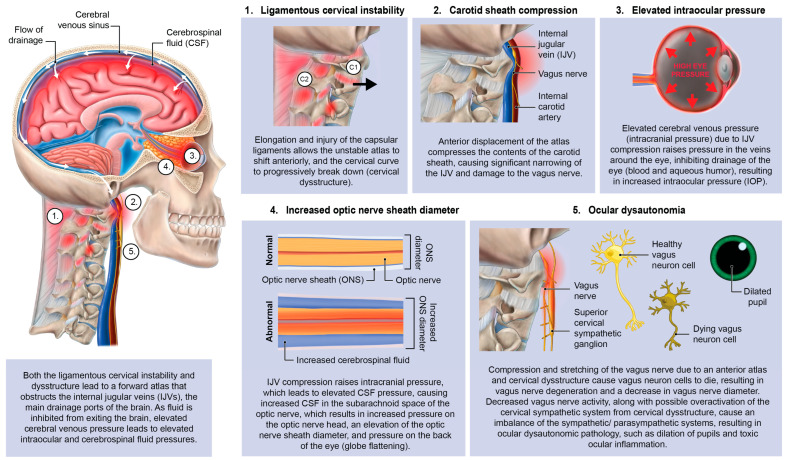
Ligamentous upper cervical instability with possible resultant eye pathology.

As discussed, an increase in CSF pressure leads to a corresponding rise in pressure within the optic nerve sheath, transmitting this increased pressure to the nerve head [211]. When CSF pressure builds up behind the eye, the optic disc tissue exhibits greater distensibility compared to the adjacent sclera, allowing CSF to swell into the cavity of the eye [211]. This rise in extraocular pressure can stimulate a rise in intraocular pressure. The lamina, situated where the optic nerve fibers (axons of retinal ganglion cells) exit the eye, features perforations in the sclera. This lamina represents the most vulnerable point in the pressurized eye wall [252]. This is a very intricate part of the visual system, where increased brain pressure and CSF pressure can exhibit a posterior eye squish that can cause damage affecting any of the structures in this area. When combined with elevated IOP, both possibly due to unresolved IJV compression, the eye may undergo “double eye squish” with pressure accumulating within the eye and posteriorly. This and other consequences of LCI can lead to resultant eye pathology (Figure 16). It is plausible that resolution of IJV compression and CSF flow may reduce intracranial pressure, IOP, and CSF pressure within the optic nerve sheath in these cases, thereby reducing the pressure and subsequent damage of a double eye squish, which could include a breakdown in the blood–ocular barriers and degenerative damage to the retina, macula, and lens. A “double eye squish” may be a rare occurrence, but its possible significance warrants further investigation, especially in cases suspected to have underlying LCI and CD.

## 13. Ocular Dysautonomia—Superior Cervical Sympathetic Ganglion Hyperactivity

**Figure 17:**
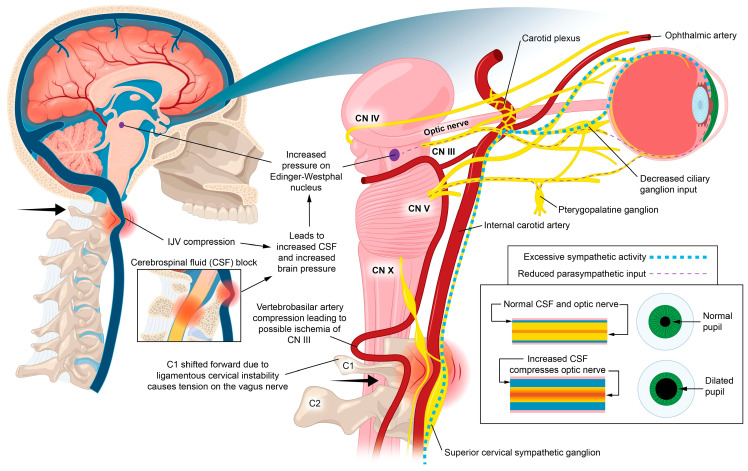
Proposed hypothetical ocular dysautonomia with pupillary dilation: Ligamentous cervical instability can lead to internal jugular vein compression and/or ocular dysautonomia, with resultant pupillary dilation.

The autonomic nervous system plays an intricate role in ocular homeostasis and visual acuity, including regulation of IOP, pupil diameter, accommodation, refraction, tear formation, and arterial blood supply [38,253,254]. The parasympathetic and sympathetic systems must synergistically balance each other. Should the system be out of balance, autonomic dysfunction could be a potentially overlooked cause of many ocular manifestations, such as myopia, mydriasis, hyperopic defocus, and transient vision loss. Ocular dysautonomia may have a cervical venous and/or nervous system etiology when underlying LCI and CD is present (Figure 17).

Parasympathetic innervation of the eye includes the Edinger–Westphal nucleus to the ciliary ganglion pathway and the superior salvatory nucleus through the pterygopalatine ganglia. Sympathetic innervation of the eye ascends through the superior cervical sympathetic ganglion (SCSG) [255]. The sympathetic visual system, in control of the elevating the eyelids and dilation of the pupil (mydriasis), starts in the hypothalamus, where fibers are set through the brainstem to the cervical spinal cord and upper thoracic spinal cord (C8-T2), and second-order neurons make their way out of the spinal cord to the SCSG [255]. Superior cervical sympathetic ganglion neurons project to the dilator pupillae muscle of the iris to control pupil dilation. The SCSG sits just in front of the second and third anterior cervical vertebrae, posterior to the carotid sheath, and anterior to the longus colli muscle. The nodose ganglion of the vagus nerve sits directly anterior to the atlas, so both the vagus and sympathetic nerves that traverse the anterior neck can be easily compressed or stretched from LCI and CD. FHP is known to cause weakening and thickening of the longus colli muscle, which could be another avenue by which changes in neck structure could affect autonomic nervous system function to the eye [256,257].

**Figure 18:**
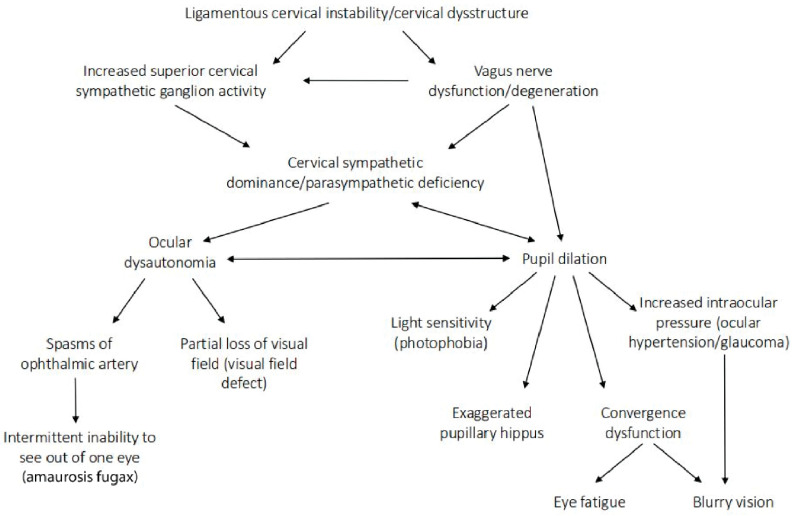
Potential (hypothetical) ligamentous cervical instability etiology of eye symptoms and diseases through superior cervical sympathetic ganglion hyperactivity.

**Figure 19:**
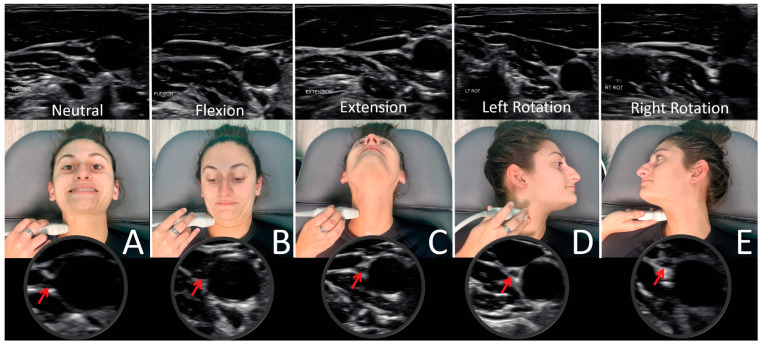
Ultrasound of vagus nerve (red arrows) in mid-cervical region with various neck positions. (**A**) Neck in neutral position. (**B**) Flexed neck. (**C**) Neck extended. (**D**) Neck rotated left. (**E**) Neck rotated right. As can be seen, the vagus nerve within the carotid sheath undergoes various structural tensions depending on neck positions.

Some mechanisms affecting the autonomic nervous system involving the eye with underlying LCI or CD might include increased CSF pressure around the cranial nerves and Edinger–Westphal brainstem ganglion, cervicovagopathy (vagus nerve degeneration from cervical pathology), sympathetic dominance by effects on the SCSG and nerves, and trigeminal nerve dysfunction secondary to effects on the trigeminocervical nucleus [258,259,260]. SCSG stimulation might indirectly cause pupil dilation, as well as lead to arterial spasms or accommodative dysfunction, though these ideas are speculative and serve to extend the primary hypothesis [261,262] (Figure 18). Sympathetic dominance can cause the pupils to dilate, retinal blood supply to constrict, and IOP to rise, though the most common symptom produced is light sensitivity (photophobia) [263]. The SCSG has been implicated in many conditions and symptoms that include elevations of intraocular pressure, glaucoma, photophobia, and macular degeneration [264,265]. It may be that this sympathetic dominance would occur if the normally inhibitory function on the sympathetic system of the vagus nerve were diminished, as the vagus nerve has direct branches to the SCSG [34,266]. The vagus nerve, running right next to the IJV, also undergoes stretch and compression forces with various neck and head motions and positions; thus it is prone to not only conduction blocks but also degeneration secondary to LCI and CD (Figure 19).

The vagus nerves are relatively small compared to their level of importance. The numbers of neurons in the vagus nerves, eye (retinal ganglion), enteric nervous system, and spinal cord are approximately 100,000, 1 million, 400–600 million, and 1 billion, respectively [267,268,269]. Another important point is that in the cervical spine, the cross-sectional area of the spinal cord is 110–120 mm^2^ and the vagus nerve is 2.0–2.5 mm^2^ [270,271]. Vagus nerve dysfunction could be the reason why so many patients have many other symptoms besides visual disturbance and neck pain, including gastrointestinal issues, dizziness, anxiety, tachycardia, and a host of others, which could differentiate the need to consider cervical vagopathy in the differential diagnosis of atypical symptom profiles.

Both the VN and SCSG are not only interconnected with each other, but also with many other structures at the CCJ. In the upper cervical region, the vagus neurons connect with the trigeminal, facial, glossopharyngeal, spinal accessory, and hypoglossal nerves (cranial nerves V, VII, IX, XI, and XII, respectively), along with the connections to the cervical sympathetic trunks and C1–C3 spinal nerve roots [272,273]. The SCSG also connects with numerous organs, vessels, muscles, bones, joints, the last 4 cranial nerves, vertebral plexus, vagus nerve, facial ganglion, phrenic nerve, middle sympathetic ganglion, pineal gland, vagus nerve, and importantly, the cerebral blood vessels [274,275].

The eye and the eye area, including the orbit, receive blood flow from several branches of the ophthalmic artery, which is the first branch off of the internal carotid artery, susceptible to vasoconstriction due to tight muscles in the cervical spine or an imbalanced ANS response. Vasculature of the eye is innervated by vasodilatory fibers from the pterygopalatine ganglion, and by vasoconstrictive fibers from the superior cervical sympathetic ganglion, meaning activation of the SCSG can cause vasospasms, which can be a cause of visual changes and eye pain due to a lack of blood flow to the ciliary muscle [276,277]. While the blood flow is controlled by parasympathetic, sympathetic, and local trigeminal nerves, the sympathetic input from the SCSG is crucial for controlling pupil dilation and mediating choroidal vasocontraction [278]. Pupillary constriction and dilation are controlled by a balance between the parasympathetic and sympathetic systems, dependent on vagal tone [279]. Underlying sympathetic dominance might contribute to a chronically dilated pupil, limiting the fluid flow out of the eye by blocking aqueous humor drainage and increasing eye pressure, as seen in glaucoma [280,281]. Diminished ocular blood flow can cause symptoms of blurry vision, usually in one eye, or partial or complete loss of vision [282].

Vasospasms are linked to visual field changes, and are even suggested to be a factor in developing low-tension glaucoma [283]. Other possible consequences of ocular vasospastic syndrome include corneal edema, retinal arterial and vein occlusions, choroidal ischemia, amaurosis fugax, and anterior ischemic optic neuropathy [284]. It has been proven that treatment of ocular vasospasm can improve or relieve visual field defects, but it is not often known what is causing them [285]. Stimulation of the vagus nerve in a triple-blind study is shown to reduce radiographic vasospasm and mitigate inflammatory response [286]. Cervical and auricular vagus nerve stimulation has been studied in relation to protecting the retina against pro-inflammatory mechanisms and preventing retinal ganglion cell loss and retinal dysfunction after ischemia, and has been shown to be safe and effective [61]. This evidence supports the notion that the vagus nerve activity is an important neuroprotective mechanism for all of the organs, especially the eyes, and the necessity to maintain or restore vagus nerve health for long-term protection. Aligning with this hypothesis, cervicovagopathy due to LCI and/or CD should be considered as an underlying etiology leading to eye symptoms, especially when automimic dysfunction may be involved [56].

## 14. Stages of Eye Degeneration Due to LCI

**Figure 20:**
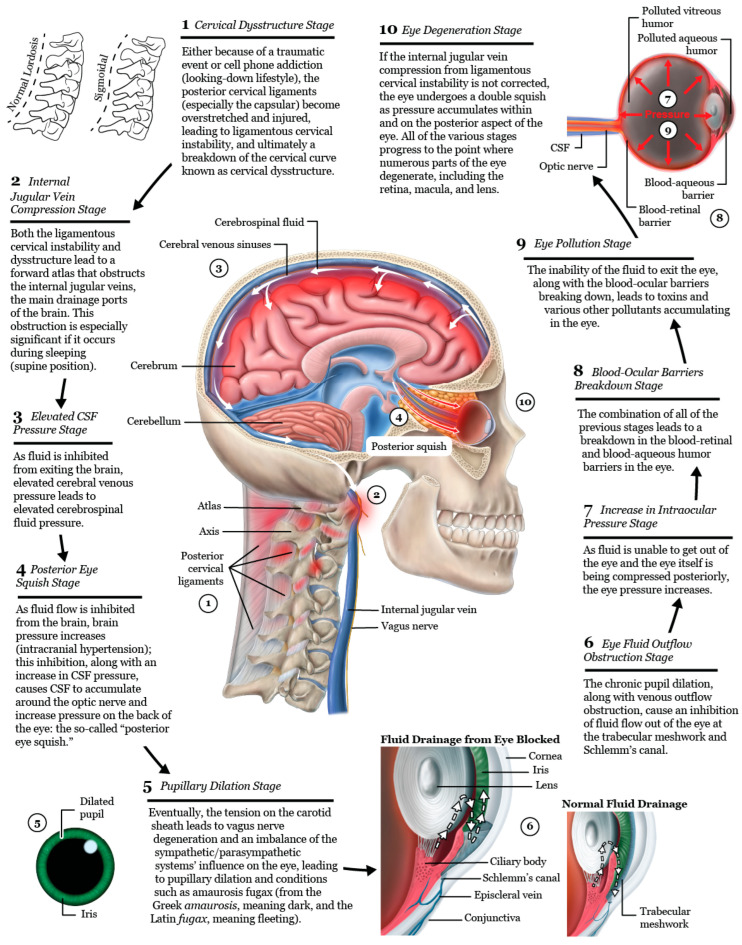
The 10 stages of eye degeneration following ligamentous cervical instability and/or cervical dysstructure (hypothesis).

LCI is a progressive disorder, and cervical oculopathy likely is as well [287]. This raises the possibility that some pathological processes which lead to serious visual symptoms and even visual field defects or visual loss from such conditions as macular degeneration and retinopathy in people without a history of diabetes or hypertension could have a structural neck etiological basis. As the breakdown of the cervical curve from ligament injury shifts the atlas forward, serious detrimental effects on fluid flow into and out of the brain from IJV compression can occur. As vagus nerve input is inhibited, sympathetic dominance in ocular autonomic nervous system physiology can occur. If flow is inhibited from both the brain (IJV compression) and the eye (potential IJV compression and chronic pupil dilation effects on Schlemm’s canal), both the brain and eye pressures increase. Eventually, metabolic waste products (inflammatory toxins and protein aggregates) will start to accumulate inside the brain and eye [288]. As with most age-related conditions—cataract, macular degeneration, retinopathy, and presbyopia—are some main eye diseases with underlying protein aggregation contributing to their development [289,290,291] (Figure 20). LCI may be considered an underlying etiology for many eye diseases, especially when the underlying cause remains unidentified.

## 15. Cervical Structural Treatment for Eye Conditions?

**Figure 21:**
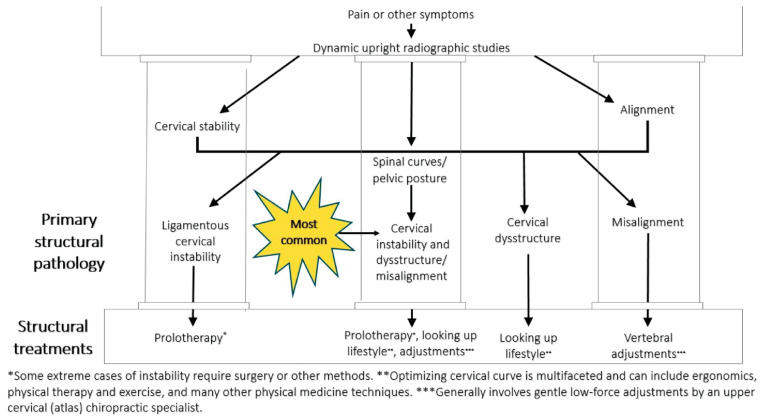
Cervical treatment recommendations based on dynamic upright radiographic studies. Patients often have a combination of ligamentous cervical instability, cervical dysstructure (breakdown of cervical curve), and misalignments, the 3 pillars of cervical structural health [96].

Ideal structural integrity of the cervical spine involves 3 primary parameters: alignment, curve, and stability. LCI can result in misalignment or subluxation, curve deformation, and progressive ligamentous instability. Dynamic structural medicine explains how human posture and movement give health or disease to the body depending on bony structural integrity. The discussed cervical structural analysis, along with the neck vitals (pathophysiologic tests), can be utilized to monitor the progress of targeted treatments of neck reconstruction therapy directed at the cervical vertebral malalignments, cervical curve dysstructure (breakdown), and instability (Figure 21).

**Figure 22:**
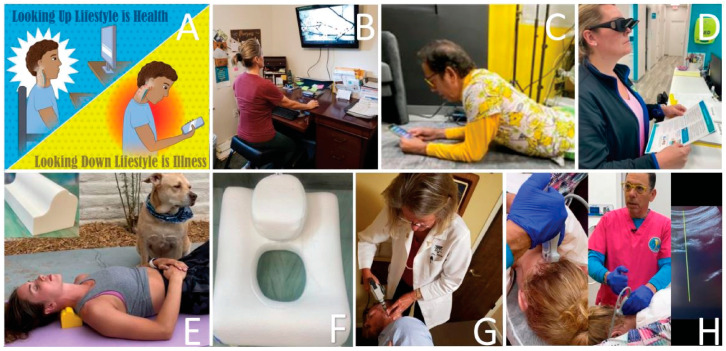
The many potential ways to improve cervical structure. (**A**) Live a “looking-up lifestyle.” (**B**) Elevate your computer screen. (**C**) Use postural cervical muscles while on cell phone. (**D**) Wear prism glasses. (**E**) Lie on a Denneroll^®^. (**F**) Sleep on a cut-out pillow. (**G**) Get an orthospinology atlas adjustment. (**H**) Receive Prolotherapy [13,90,256,292,293].

**Figure 23:**
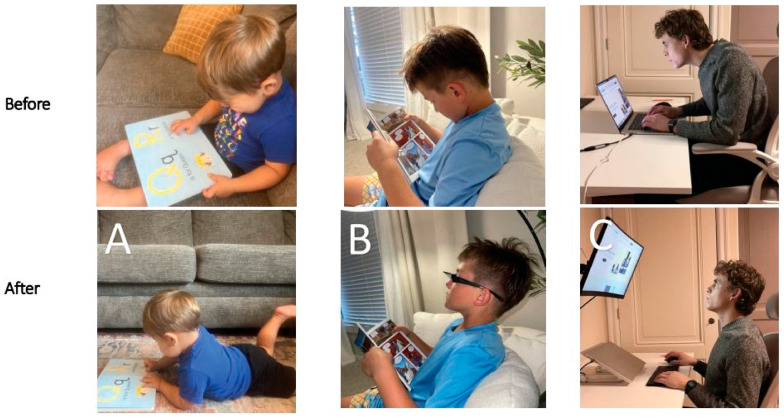
Lifestyle modifications in which cervical curve postures can possibly be improved. (**A**) Lying prone. (**B**) Wearing prism glasses. (**C**) Raising computer height.

If brain, eye, vision, and additional symptoms are related to changes in the cervical curve that occur because of an FDFH lifestyle by the slow stretching of the cervical ligaments, then resolution of such symptoms might occur with therapies such as physical therapy, therapeutic exercise, postural ergonomic changes when looking at electronic devices, gentle chiropractic or osteopathic adjustments, and therapies that assist in restoration of the cervical curve and its stabilization by tightening of the ligaments with Prolotherapy [13,90,256,292,293] (Figure 22). Perhaps the most important way to improve cervical structure is to have a natural “looking up” lifestyle, even in young children, while using computers and cell phones. See Figure 23.

**Figure 24:**
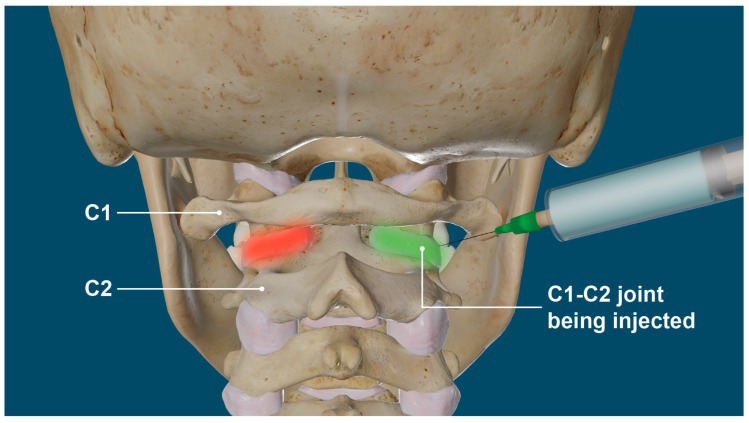
Prolotherapy to C1–C2 facet joints. By stimulating capsular ligament repair, Prolotherapy tightens the facet joint, resolving upper cervical instability (red area) and its related symptoms.

Prolotherapy was developed by George Hackett, MD in the 1950s with the primary target to treat potential pain sources within connective tissue by either proliferation of new cells or the improvement in the health of existing cells [294,295]. Evidence that Prolotherapy induces the repair of ligaments and other soft tissue structures, including tendons, has been reported in both animal and human studies [296,297,298]. Prolotherapy injections in the neck are directed at the posterior ligament complex, especially the capsular ligaments surrounding the facet joints, with the goal of tightening them to resolve the LCI (Figure 24). Prolotherapy to the cervical spine involves injecting solutions of dextrose, polidocanol, platelet-rich plasma, and other solutions primarily to the facet capsular ligament attachments at the bony interface, so in that regard, the most important injections of Prolotherapy are periarticular (not intra-articular), since the instability is from ligament dysfunction or injury [294,299]. Prolotherapy in the upper cervical region has to be done with extreme care because of the vertebral arteries’ proximity to the upper cervical facet joints, so the procedure is done under ultrasound guidance. Repeat cervical structural evaluations such as DMX can be utilized to document successful cervical lordosis curve restoration and stabilization.

## 16. Limitations and Future Directions

Current standard treatments for eye diseases range from semi-invasive to invasive options such as medications, laser procedures, intraocular injections, and surgeries. There is a current lack of large prospective, randomized, or interventional studies correlating structural neck issues with eye diseases and symptoms, meaning the proposed links remain theoretical. There are methodological challenges in studying the proposed mechanisms, as there is not yet standardization of what constitutes ligamentous upper cervical instability, especially regarding the facet joints, optimal cervical lordotic curve, forward head posture, excessive pupillary dilation, vagus nerve degeneration, and dynamic internal jugular vein compression. Comparing the suggested parameters on people with visual symptoms in this paper to those without would add credibility to the proposed mechanisms. Future prospective studies will need to demonstrate a correlation of improved neck structural dynamics with ultrasound measurements of the VNs and IJVs, along with improved visual/eye symptoms, to give credence to the mechanisms discussed. Future research should develop and implement standardized protocols to evaluate the discussed mechanisms, including dynamic structural imaging, quantitative pupillometry, and hemodynamic assessments. Including functional imaging studies involving fluorescein angiography or optical coherence tomography, documenting retinal blood flow, cellular health, and biomarkers with the parameters discussed here, would further clarify the relationship between dynamic neck structure and retinal health or disease.

Most chronic eye conditions are progressive and often occur in an older population in the context of systemic comorbidities such as diabetes. When eye and visual symptoms and conditions start to present in a younger and healthier population, a cervical structural etiology should be considered as a potential underlying cause. Abnormal structural neck, VN, and IJV parameters may just be an association and not causation. Alternative explanations for visual symptoms can include previously undiagnosed autoimmune disease, nutritional deficiencies, neurological disorders, medication side effects, refractory errors, coagulopathies, metabolic conditions, genetic issues, and a host of others [300,301].

## 17. Conclusions

This paper explores potential mechanisms, including neurovascular and neurologic pathways that originate in the cervical spine, which may underlie many eye and visual conditions. Considering the rise in text neck and computer vision syndrome, along with many other visual conditions, the modern lifestyle of excessive FDFH posture is contemplated as a cause for the slow stretching of posterior ligaments (termed “creep”) in the cervical spine, especially the capsular ligaments, that results in LCI and cervical dysstructure, promoting a forward-shifted atlas that causes carotid sheath compression. It is hypothesized that cervical structural dysfunctions, LCI, and cervical dysstructure lead to: (1) impaired venolymphatic drainage of the eye and brain by compression of the internal jugular veins, and (2) ocular dysautonomia from a disruption in the parasympathetic/sympathetic system balance, in part from vagus nerve degeneration. To support this hypothesis, proposed potential effects on the visual system include functional and mechanical stress on the eye, such as altered hemodynamics and hydrodynamics, and ocular dysautonomia. The functional abnormalities can result in an elevation of cerebrospinal fluid pressure increasing eye and brain pressures and fluid around the optic nerve head, ultimately putting the eye under structural stress such as elevated IOP, a “double eye squish” with resultant breakdown of the blood–ocular barriers, and a “toxic” neurodegenerative-inducing environment, as well as autonomic imbalance, including chronic pupillary dilation.

Many distressing visual and ocular symptoms occur from elevated brain and eye pressures, nervous system dysregulation, and other disorders which affect eye physiology. The catalyst for many conditions with an otherwise unidentified etiology may be LCI and cervical dysstructure. LCI can be documented by dynamic upright digital motion (fluoroscopic) analysis. The pathology it initiates can be documented by a testing protocol known as neck vitals, which includes cervical and ocular ultrasound exams, transcranial and extracranial Doppler ultrasound, pupillometry, and tonometry. If underlying cervical spine abnormalities are confirmed, treatments can be directed at cervical vertebral misalignments, cervical lordotic curve breakdown, and LCI by gentle adjustments, a “looking up” lifestyle, directed exercise, physical therapy, and Prolotherapy. Prolotherapy injections—a regenerative therapy—induce the strengthening and tightening of the injured dysfunctional ligaments, promoting cervical spine stability. The improvements in patients’ symptoms and diseases, and their structural anatomy and integrity, can be objectively documented, along with the pathology they cause, by serial dynamic radiographic/ultrasound and neck vital analyses. If the eye symptoms or diseases are caused by LCI and cervical dysstructure, then treatments aimed at correcting cervical structure and improving stability should improve—or even resolve—symptoms previously attributed to primary ocular conditions. This framework provides a mechanistic hypothesis by which cervical spine dysfunction can contribute to visual symptoms and eye diseases, a relationship that future studies should aim to clarify in order to advance diagnostics, treatment, and clinical outcomes for patients with otherwise unknown etiology.

## Data Availability

The original contributions presented in this study are included in the article. Further inquiries can be directed to the corresponding author.

## Abbreviations

The following abbreviations are used in this manuscript:

ANS	Autonomic nervous system
BRB	Blood–retinal barrier
C6AI	C6-atlas interval
CBCT	Cone beam computed tomography
CCJ	Craniocervical junction
CD	Cervical dysstructure
CNS	Central nervous system
COP	Cervical oculopathy
CSA	Cross-sectional area
CSF	Cerebrospinal fluid
CT	Computed tomography
CVS	Computer vision syndrome
DOC	Depth of curve
DMX	digital motion X-ray
FDFH	Facedown/forward head
FHP	Forward head posture
hEDS	Hypermobile Ehlers-Danlos syndrome
ICH	Intracranial hypertension
ICP	Intracranial pressure
IJV	Internal jugular vein
IOP	Intraocular pressure
LCI	Ligamentous cervical instability
MRI	Magnetic resonance imaging
ONH	Optic nerve head
ONSD	Optic nerve sheath diameter
SAS	Subarachnoid space
SCSG	Superior cervical sympathetic ganglion
TCD	Transcranial Doppler
TLPD	Translaminar pressure difference
TNS	Text neck syndrome
